# Seed Cryopreservation and Germination of *Rhus glabra* and the Critically Endangered Species *Rhus michauxii*

**DOI:** 10.3390/plants10112277

**Published:** 2021-10-24

**Authors:** Gerald S. Pullman, Kylie Bucalo, Ron O. Determann, Jennifer M. Cruse-Sanders

**Affiliations:** 1 Renewable Bioproducts Institute, Georgia Institute of Technology, 500 10th Street NW, Atlanta, GA 30332, USA; kbucalo@ohanainstitute.org; 2 School of Biology, Georgia Institute of Technology, Atlanta, GA 30332, USA; 3 Department of Conservation Research, Atlanta Botanical Garden, 1345 Piedmont Ave., NE, Atlanta, GA 30309, USA; rondetermann@gmail.com (R.O.D.); crusesanders@uga.edu (J.M.C.-S.); 4 State Botanical Garden of Georgia, University of Georgia, 2450 Milledge Avenue, Athens, GA 30605, USA

**Keywords:** conservation, cryopreservation, endangered species, micropropagation, shoot culture, *Rhus michauxii*, dwarf sumac, Michaux’s sumac, false poison sumac

## Abstract

*Rhus michauxii* is a perennial rhizomatous shrub native to the southeastern United States that is found mainly in sunny, dry, open rocky or sandy woodlands. Moreover, it is found on ridges or river bluffs in the inner coastal plane and lower piedmont of Virginia, Georgia, and the Carolinas. Habitat conversion to agriculture, suppression of fires, and low reproduction have caused *R. michauxii* to become rare and it is now federally listed as threatened. Methods are needed to multiply and conserve *R. michauxii*. Protocols were developed for seed cryopreservation, in vitro germination, and micropropagation for *R. glabra* and *R. michauxii*. Seed scarification in concentrated sulfuric acid for 6 h and germination on ½ MS medium resulted in germination up to 96% for control and cryopreserved seeds of *R. glabra* and 70 and 40% for control and cryopreserved seeds of *R. michauxii*. Shortly after germination in vitro, young seedlings were established in a greenhouse potting mix providing new plants from the endemic Georgia *R. michauxii* populations. Several of the findings meet goals within the *R. michauxii* recovery plan by providing methods for sexual and asexual multiplication and long-term seed storage under cryogenic conditions. The protocols developed will assist in the safeguarding and conservation of dwindling natural *R. michauxii* populations.

## 1. Introduction

*Rhus michauxii* Sargent is a rhizomatous perennial shrub that belongs to the Anacardiaceae family. Commonly known as Michaux’s sumac, dwarf sumac or false poison sumac, *R. michauxii* is a federally listed threatened plant endemic to the southeastern US [1]. Michaux’s sumac is found in sunny, dry, open, rocky or sandy woodlands over mafic bedrock. In addition, it is often found on ridges or river bluffs in the inner coastal plane and lower piedmont of Virginia, Georgia, and the Carolinas [2]. *Rhus michauxii* is dioecious. The male and female plants respond differently to vegetative competition, contributing to the presence of only one sex in most of the extant populations. As a result, they reproduce only clonally [3]. Habitat conversion to agriculture and fire suppression that allow the invasive plant species to displace Michaux’s sumac have caused populations to become fragmented, smaller or extirpated altogether over the past few decades. Since the species was discovered in 1796, almost 50% of the known populations have been extirpated [4].

Michaux’s sumac has about 54 extant populations remaining [3]. These populations are most often geographically isolated from each other by large distances. In addition, most of them contain only plants of a single sex, representing a major obstacle to sexual reproduction in its natural habitat. In Georgia, this species is rated as S1, critically impaired [5]. Most of the extant populations are in North Carolina, with two identified in Georgia and six in Virginia [3]. Previous populations known in Florida and South Carolina are thought to be extirpated and the species appear to be disappearing from the southern half of its range [6]. Genetic studies show a low amount of genetic diversity in the remaining populations, indicating another possible roadblock to recovery, as low genetic variability corresponds to a lower plasticity in the response to environmental selection pressures [7,8].

The Endangered Species Act of 1973 directed several subsidiary agencies to follow recovery plans to promote the conservation and protection of endangered or threatened species. Each recovery plan specifies research goals and the management actions needed to support recovery and reintroduction of an endangered species. The federal recovery plan for *R. michauxii* calls for the protection and management of all the extant populations to ensure their survival [4]. Collection of seed and or vegetative propagules for ex situ conservation is needed, along with research into seed dispersal and germination requirements [4].

In vitro techniques have been useful to germinate and multiply endangered plants asexually [9,10,11]. Micropropagation has many advantages, including rapid production of multiple clones of the same plant. In vitro generated plants often display lower rates of disease and contamination, as well as more robust or accelerated growth in comparison to members of the same species grown from seeds or cuttings in soil. Therefore, members of endangered species produced from micropropagation stand to be of great benefit in supplementing small populations or to establish new populations in suitable locations for conservation, research, educational or recreational purposes.

Cryopreservation has been used for seed and shoot tip conservation [11,12,13,14,15]. In addition, it is considered to be the best long-term approach to preserving endangered species [12]. Conservation of this species is important not only for its ecological value, but also for its medical potential. Multiple species in the genus *Rhus* have shown their medical value including antibacterial, antifungal, antimalarial, anticancer, and other medicinal properties [16,17,18,19]. Fruit from *R. glabra* or *R. typhina* fed to horses can even help control chronic emphysema [20]. Unfortunately, due to its rarity, *R. michauxii* has not been investigated for bioactive compounds other than to indicate that it is not poisonous [21]. 

The members of the Anacardiaceae family require that dormancy of the hardened seed coat should be removed physically so the water can enter the seed and stimulate germination [22]. Common methods to remove physical dormancy include: Mechanical scarification where the seedcoat is nicked or disrupted, chemical scarification in concentrated acids to partially dissolve the seed coat and simulate passage through the digestive system of an animal, short-term high temperature treatments including short-term submersion in boiling water for minutes to hours at temperatures near or above 100 °C, short-term fire exposure, and long-term exposure to heating and cooling [23]. A limited amount of literature on *R. michauxii* makes it difficult to identify the best approaches to germinate seeds for this species. Other sumac species have been successfully scarified with sulfuric acid, suggesting that a sulfuric acid treatment may work for *R. michauxii* [22,24,25,26,27]. Dumroese et al. [27] suggested that seed coat hardness during scarification can be examined by manually opening a few seeds at regular intervals to determine if the seed coat was softer than it was before. With microscopic observations of coat thickness and damage, this crude tool can help evaluate if the scarification time is too short or too long.

Our objectives were to develop a reliable system of long-term seed or shoot-tip cryopreservation and micropropagation to preserve *R. michauxii*. In 2010, when our study began, there were no reports of *R. michauxii* germination in the published literature. In order to physically damage the seed coat and simultaneously surface-sterilize seeds for germination in vitro, a scarification treatment in concentrated sulfuric acid that is used to germinate several other *Rhus* species was selected [22,24,25,26,27,28]. Through a series of experiments, three approaches to conservation were tested: Germination of seeds to produce plants for in situ or ex situ collections, micropropagation of shoots for plant production, and cryogenic storage of seeds. Due to the lack of available seeds for experimentation early in the study, seeds of *Rhus glabra* were used to help develop a preliminary system. *R. glabra*, known as smooth sumac, is commonly found throughout the lower 48 states of the US. *R. glabra* is very similar to Michaux’s sumac with a thick seed coat. We hypothesized that the close relationship between these species would allow for the development of a germination and micropropagation system that could later be adjusted for *R. michauxii*.

## 2. Results

### 2.1. R. glabra Seed Sterilization and Germination Experiments

**Figure 1:**
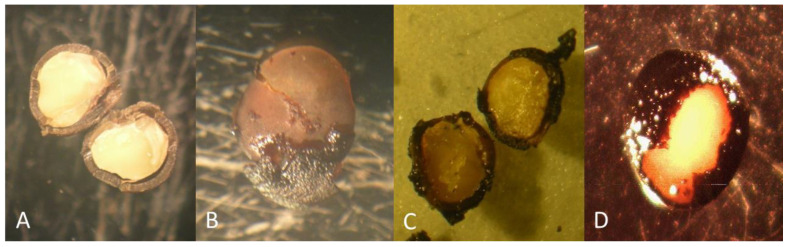
*Rhus glabra* seeds after scarification. (**A**) No scarification. (**B**) 2 h. (**C**) 4 h. (**D**) 7 h.

**Figure 2:**
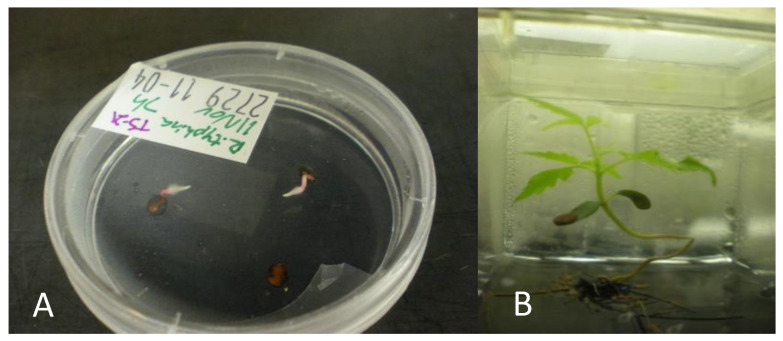
*Rhus glabra*. (**A**) Germination in vitro. (**B**) Seedling growth in vitro.

**Figure 3:**
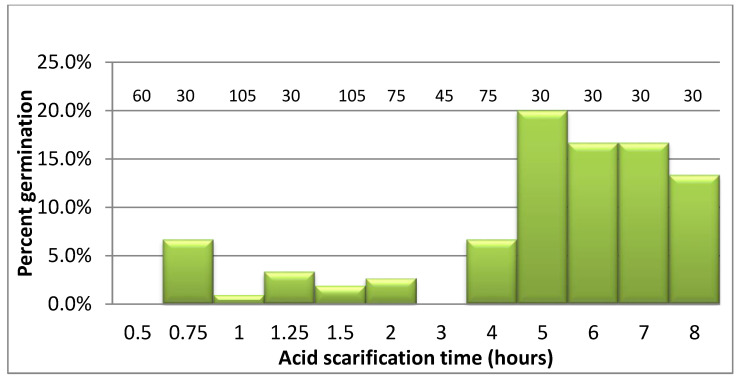
*Rhus glabra* germination after scarification in concentrated sulfuric acid. The number of seeds tested per scarification time is shown above the bar. Germination results per time are combined from several germination media.

Observations of cut seeds showed a thick coat prior to scarification (Figure 1A). After 2–4 h in sulfuric acid the coat was still solid, and by 7 h the damage was visible (Figure 1B–D). Germination occurred in most of the scarification treatments (Figure 2), but was maximized at 5–7 h. Germination results from four experiments including 645 seeds are combined for each scarification time in Figure 3. Seeds began to germinate after 1 week, reaching maximal percentages by 1 month. The shorter scarification time resulted in the higher contamination and less germination. After 4 h, fewer seeds became contaminated. Germination, defined as the emergence of a radicle, was similar among media. However, seedlings appeared to be more vigorous in media containing the woody plants medium (WPM) and root growth was impacted negatively by BAP. BAP induced the growth of a single, thick long root rather than many lateral roots present in media without BAP. When germination percentages were combined for scarification times of 5–8 h, germination for ½ MS, MS, and WPM, each with 0.5 mg/L BAP, was 10, 12, and 18%, respectively.

Twenty-one of the 180 *R. glabra* seeds floated and were discarded. Most of the *R. glabra* seeds germinated in 10 days. Germination was 40/52 (77%) for ½ MS, 49/52 (94%) for MS, and 48/52 (92%) for WPM. Three seeds were contaminated with one per treatment. Seedlings in WPM appeared more robust during early germination, but after 30 days all of the seedlings appeared similar. Differences between the treatments were statistically significant at *p* = 0.01.

### 2.2. R. michauxii Seed Scarification, Germination, and Seedling Establishment in Potting Mix

**Figure 4:**
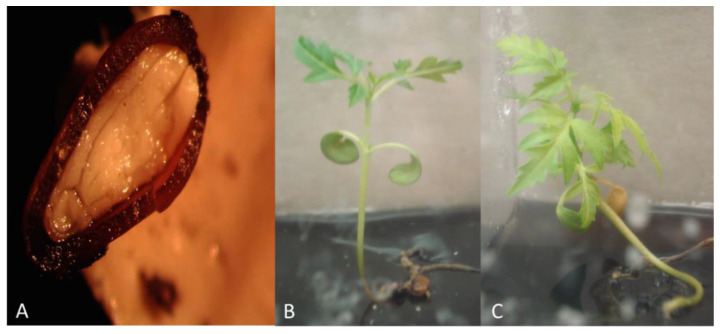
*Rhus michauxii* seeds (Source 1) and seedlings from 2005 from the North Carolina Botanical Garden. (**A**) Non-scarified seed showing the thick endocarp and true seed coat and embryo inside. (**B**,**C**) Two resulting seedlings from seeds scarified for 6 h and germinated in vitro on ½ MS medium without hormones.

The *R. michauxii* seed coat thickness was similar to *R. glabra* (Figure 4A). The scarification protocol developed for *R. glabra* worked well for *R. michauxii* seeds (Source 1) and germination occurred with little contamination. Ninety-three percent of the seeds were empty as indicated by dissection after 2 months without germination. Eleven of the 148 seeds contained embryos. Of these three seeds germinated, one began to germinate but died, six did not germinate and showed damage of over scarification, and one was used to evaluate the coat thickness. Seedlings grew on the germination medium for several weeks (Figure 4). However, attempts to micropropagate the surviving seedlings on the medium used for mature *R. michauxii* tissue (MS + 0.05 mg/L BAP) did not succeed. Shoot tips and seedling bases stopped growing, slowly deteriorated, and died.

**Figure 5:**
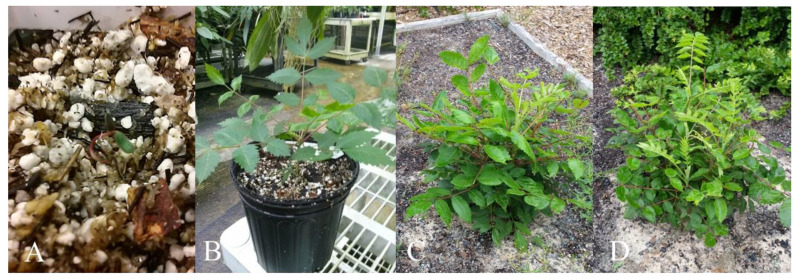
*Rhus michauxii* germinants from Source 2 seeds collected from the Lower Broad River Wildlife Management Area in 2012. (**A**) Seed germinated in vitro and transferred to soil when the radicle first appeared. Seedling is shown emerging from the soil. (**B**) Seedling after 1 year in the greenhouse. (**C**,**D**) Two seedlings germinated in vitro grown in the greenhouse for 1 year and outside for another year. Note the new stem formation.

Of the 114 *R. michauxii* seeds (Source 2), 63% sank in water and 37% floated. Seven percent of the floaters were contaminated and 2/26 non-contaminated seeds germinated. Nineteen percent of the sinkers were contaminated and 2/32 non-contaminated seeds were germinated. All of the four germinants were planted in greenhouse soil shortly after germination and established young seedlings. Two continued to grow, establishing plants in the greenhouse that later survived transplanting to a safekeeping site (Figure 5). These plants represent the first known new plants from an endemic Georgia *R. michauxii* population. The scarified seeds planted directly in the potting mix did not germinate.

**Figure 6:**
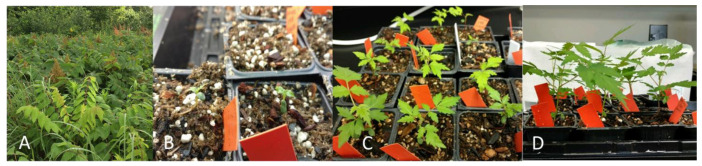
(**A**) *Rhus michauxii* male and female plants flowering at the Atlanta Zoo rooftop garden. (**B**) Seedlings from 6-h-acid-scarified seeds germinated in vitro and transferred in the potting mix 2 days after the radicle became visible. (**C**) One month after planting. (**D**) Seven weeks after planting.

Germination and plant establishment were tested for three seed sources in 2018. *R. michauxii* seeds (Source 4) had the highest percentage of filled seeds and about 40% of these germinated producing 16 seedlings that were transferred in the potting mix (Table 1). Seeds from Sources 5 and 6 (Figure 6A) showed low percentages of filled seeds (Table 1). One of eight Source 6 seeds germinated and produced a seedling (Table 1). Seed contamination in vitro for Sources 4 and 5 was 26 and 13%. Of the 23 germinants, 17 were planted in the potting mix and grew well, two were weak, and three died (Figure 6B–D).

### 2.3. R. glabra Shoot Culture Experiments

**Figure 7:**
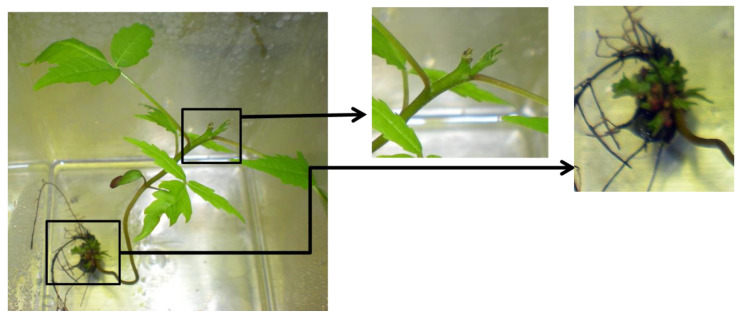
*Rhus glabra* seedling basal portion in multiplication medium. Note: New axillary bud growth at the cut stem and basal shoots from the rhizome.

Shoot tips and basal segments formed new shoots from axillary buds, and new shoots grew at the shoot/root junction forming a microrhizome (Figure 7). In the first experiment, shoot production from intact plants was 2.5 new shoots per shoot compared to 3.0 new shoots per shoot with tip removal. Differences were not statistically different. In the second test, dark cold-grown shoots produced 4.0 new shoots per shoot vs. 1.8 for shoots grown in the light. Differences between the treatments had a significance value of *p* = 0.06. In the third test, shoot production on the medium with activated charcoal (AC) without PGRs was 0.2 new shoots compared to 1.3 on the medium with AC and BAP. Differences were statistically significant at *p* = 0.03.

### 2.4. R. michauxii Bud Sterilization and Multiplication Experiments

**Figure 8:**
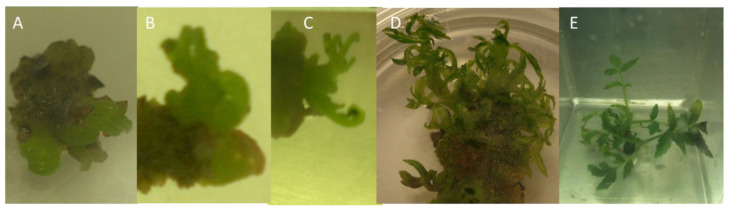
Sequence of growth for one bud taken from an outdoor-grown *Rhus michauxii* female plant. (**A**) Multiple buds appearing on the bud after 2 weeks on ½ MS + 0.5 mg/L BAP. (**B**) Four weeks on ½ MS + 0.5 mg/L BAP. (**C**) Five weeks on ½ MS + 0.5 mg/L BAP. (**D**) Multiplying shoots after 3 months on MS + 0.05 mg/L BAP with hyperhydric shoot appearance. (**E**) Multiplying shoots after 1 year on MS + 0.05 mg/L BAP.

In the first sterilization procedure, four greenhouse buds were not contaminated and two from 1/3 MS + 2.0 mg/L trans zeatin began to grow. All of the buds except for two collected from the outdoors were contaminated and did not grow. The second procedure removed most of the contamination, but was too severe and most of the buds did not grow. One clean bud from the outdoors, from ½ MS with 0.5 mg/L BAP began to grow and formed new buds (Figure 8A). The third sterilization procedure used only the outside buds and they all became contaminated.

With only a few explants remaining clean after sterilization, the buds were transferred to several media. The buds appeared to develop better on MS with 0.5 mg/L BAP, but the shoots did not elongate. A short subculture on the PGR-free medium showed some shoot emergence. A growth sequence for one bud is shown in Figure 8A–C. Reduction of BAP from 0.5 to 0.05 mg/L reduced bud proliferation, but facilitated shoot development. Over multiple subcultures on MS + 0.05 mg/L BAP, the shoot structure improved from hyperhydric (Figure 8D) to normal (Figure 8E). After 1 year in culture and building to several hundred shoots, the cultures began to spontaneously show presence of endophytic bacteria. Once the bacteria appeared, the cultures declined and were discarded. Within several months, all of the cultures showed the same presence of bacteria.

### 2.5. R. michauxii Root Induction In Vitro and Acclimation to Soil

All of the shoots with spontaneously-formed roots and those planted in the greenhouse potting mix slowly declined and died. The last to die were the shoots with a normal appearance.

### 2.6. R. glabra and R. michauxii Seed Cryopreservation in Liquid Nitrogen

*R. glabra* seeds had 10.2% water content. Within 7 days, 45/50 (90%) of the control seeds germinated, increasing to 47/49 (96%) within 3 weeks with one contaminated seed. Cryopreserved seeds showed 47/48 (98%) germination after 7 days and 3 weeks with two contaminated seeds. Differences in the germination percentages were not significant and indicated that *R. glabra* could survive cryopreservation.

*R. michauxii* (Source 3) seeds survived cryopreservation. When data were analyzed in a 2 × 3 factorial arrangement (±cryopreservation × three media), germination for cryopreserved seeds averaged 40% vs. 70% for non-cryopreserved seeds and the differences were statistically significant (*p* = 0.02). Germination for ½ MS, ½ MS with reduced phosphate, and ½ MS with added BAP averaged 50, 55, and 60%, respectively and the differences were not statistically significant. Control and cryopreserved seeds planted in soil did not germinate. Attempts to continue seedling growth in several MS-based shoot culture media were made, but the shoot cultures did not multiply and slowly died.

## 3. Discussion

A scarification method was developed for in vitro germination for *R. glabra* and the critically endangered *R. michauxii*. When combined with an optimized scarification time and tissue culture medium, germination percentages reached 98% for *R. glabra* and 70% for *R. michauxii*. The harsh scarification treatment (5 h or longer) killed surface-contamination and began to dissolve and damage the seed coat, leading to the removal of dormancy and germination within 1 month. Early seedlings of *R. michauxii* were transferred successfully to the greenhouse, providing the first known new plants from the endemic Georgia populations. In addition, successful seed cryopreservation protocols are reported for the first time for *R. glabra* and *R. michauxii*. Several of the findings meet goals within the *R. michauxii* recovery plan by providing methods for rapid sexual and asexual multiplication and long-term seed storage under cryogenic conditions [4].

In the absence of *R. michauxii* seed for research, we used a closely related species, *R. glabra*, with a thick seed coat, to help develop the scarification protocol. *R. glabra* and *R. michauxii* have similar morphology. In addition, Yi et al. [29] placed them together in the same subgenus *Rhus* based on sequences of internal transcribed spacers (ITS), chloroplast ndhF gene, and chloroplast trnL-F regions. Yi et al. [29] suggested that *R. glabra*, *R. michauxii*, and *R. typhina* from eastern North America and *R. coriaria* from southeastern and western Europe are closely related species.

Farmer et al. [24] compared the germination of four seed sources, each of *R. glabra* and *R. copalina* after scarification times of 10–360 min and temperatures of 5–15 °C, 10–20 °C, and 20–30 °C. The temperature had little effect on germination and seed sources varied highly. *R. glabra* required much longer scarification times compared to *R. copalina*. *R. glabra* germination maximized at 240 min of scarification and seed damage increased to 34 and 44%, respectively as the seeds were scarified for 240 or 360 min with germination at 20–30 °C. Li et al. [22,25] studied the seed morphology and physical dormancy of several North American *Rhus* species. Maximal germination occurred in *R. glabra* when the seed was exposed to boiling water from 3 s to 5 min. The rapid heating caused a blister to form adjacent to the capillary micropyle, providing an entry point for water to move through the water-impermeable endocarp. With water able to move through the seed coat, imbibition and germination approached 95% or greater. Long boiling periods killed some seeds and most of the seeds heated at 100 °C for 10 min or longer were not viable. Exposure to concentrated sulfuric acid progressively increased imbibition and germination from 24/24% with a 1-h treatment to 86/69% after 4 h. Longer exposures to the acid were not tested.

Due to the rarity of *R. michauxii* seeds, only a few attempts at germination are reported [30,31,32]. In a non-published report to the US Army, Wilkinson et al. [30] indicated up to 30% germination under continuous light after scarification with sulfuric acid for 4 h, 18% with boiling water for 3 min, less than 1% after 5–15 min of dry heat at 74–103 °C, and no germination in the control groups. Bolin et al. [31] found 62% germination on moistened filter paper for manually scarified (nicked) seeds. The dry heat treatments were applied at 60, 80, 100, 120, and 140 °C for 5 and 10 min, the control treatments did not show any germination, and the heat treatments at 100 °C for 5 min or longer killed most of the embryos. Welfare et al. [32] tested the effect of endozoochory by Northern Bobwhite Quail (*Colinus virginianus*) on *R. michauxii* seed germination in moist sand. Although 52–89% of the seeds were digested, the seeds recovered after passage through the quail digestive system showed about 17% germination compared to 1% in control seeds.

Braham et al. [33] and Emrick et al. [34] successfully transplanted *R. michauxii* shoots with roots and root cuttings and increased the number of above ground shoots. It is interesting to note that Braham et al. [33] did not find rhizomes on the roots they dug up, indicating that shoots develop adventitiously from the roots. This finding bodes well for new shoot production from roots in vitro.

In our trials with plants grown in the greenhouse and outdoors, the buds collected in October were becoming dormant and most of them did not produce non-contaminated tissue. The buds collected in November, again entering dormancy, were all contaminated after surface sterilization attempts. Our earliest tissue collections provided the few buds that we were able to decontaminate. We speculate that the buds collected in spring or early summer from the tissue with rapid growth would be easier to surface sterilize. Over time, the shoot cultures adjusted to the ½ MS medium that contained 0.05 mg/L BAP and grew at first with a hyperhydric appearance. Then, they become increasingly normal. It is not known if the endophytic bacteria occurred prior to culture and remained dormant or entered during a culture transfer, as the bacteria were not visibly present until many subcultures had occurred.

*R. glabra* shoots on the ½ MS medium with 0.05 mg/L BAP were able to multiply slowly from axillary buds and formed a microrhizome with new shoot development. *R. michauxii* germinants, however, did not continue to grow on this medium. Due to the rarity of shoots, few tests occurred with alternative media. With additional research, it is hoped that an alternative medium will facilitate *R. michauxii* shoot multiplication and microrhizome development. With the development of microrhizomes, it may be possible to plant the microrhizomes directly into the soil, as occurs with turmeric [35,36].

*Rhus michauxii* persists from year to year and spreads vegetatively by maintaining and extending underground rhizomes. Formation of *R. glabra* shoots in vitro at the shoot base indicates that the young shoot is beginning to form a rhizomataceous structure. *R. glabra* rhizome shoots were increased with both BAP and cold/dark treatments providing a natural shoot multiplication process that also may work for *R. michauxii*.

Cryopreservation of seeds, somatic embryos, plant meristems or other tissues provide an excellent tool for germplasm safekeeping [11,14,15,37,38]. The optimum moisture content for seed cryopreservation varies from 7 to 14%, depending on the species and seed lipid content [13]. Our determined seed water contents of 10.2% allowed the successful cryopreservation of *R. glabra* seeds, but due to the rarity of *R. michauxii* seeds the water contents were not measured. The responses of species to cryostorage fall into three categories: Germination increases, decreases or remains the same before and after cryopreservation. Pence [37] evaluated 237 temperate species native to Ohio and found germination reduction after cryopreservation in 21% of the species tested. Salomão [39] evaluated 66 orthodox Brazilian Savannah and Atlantic Forest Biome species from 21 botanical families and found that exposure to liquid nitrogen decreased germination in six species. Our data show survival of *R. glabra* and *R. michauxii* after cryopreservation. However, germination percentages were reduced compared to the control seeds for *R. michauxii*.

Tests showed germination in culture medium vs. no germination in soil after scarification with or without cryopreservation. It is possible that the scarification time of 6 h was too long. Due to the rarity of seeds, time in acid was based on *R. glabra* results and has not been optimized for *R. michauxii*. Excess scarification times in acid would likely damage the endosperm and embryo and allow the entry to microbes when planted in non-sterile soil. Moreover, cryopreservation may cause cracks in the seed coat that facilitate acid entry into the seed. Therefore, the cryopreserved *R. michauxii* seed may require reduced scarification times compared to the non-cryopreserved seeds. Indeed, Khanna et al. [40] showed more frequent germination in the medium than in the soil mix after cryopreservation for several *Sarracenia* species. Scanning electron microscopy showed that the liquid nitrogen treatment produced cracks and fissures in *Sarracenia* seed coats. Germination in vitro after scarification would avoid damage due to the microbes present in the soil. In addition, the medium nutrients may support and stimulate the growth of a weak or damaged embryo.

It is possible that the Smithgall seeds could be hybrids between *R. michauxii* and *R. glabra* as *R. glabra* is known to grow locally. In addition, hybridization between these two species has been reported and confirmed by allozyme analysis [8,41].

With continued habitat destruction and low frequency of burning which removes competing vegetation, loss of *R. michauxii* populations are expected to continue. Therefore, developing methods for long-term seed storage and biodiversity maintenance are critical. The procedures developed here provide tools to preserve germplasm and genetic diversity in *R. michauxii* and should be implemented immediately to assist in the conservation of these plants.

The findings in this study provide a starting point for long-term seed storage and production of *R. michauxii* plants for conservation and safeguarding (Table 2). Further studies are needed to improve, optimize, and simplify procedures to germinate *R. michauxii* seeds, micropropagate shoots, and to grow planting stock. Major needs include: (1) Optimization of scarification times for cryopreserved and non-cryopreserved seeds; (2) improvement of shoot culture; and 3) improvement of methods to transfer germinants or micropropagated shoots to the greenhouse for optimal growth.

## 4. Materials and Methods

### 4.1. Plant Materials, Experimental Design, and Evaluation

*Rhus glabra* seeds were provided from wild plants growing near the Smithgall Safeguarding Conservation Nursery near Gainesville, GA, USA in October 2010. The fruits were rubbed by hand or opened with tweezers to collect the seeds, which were allowed to dry for at least 24 h.

*R. michauxii* buds were provided by the Atlanta Botanical Garden (Atlanta, GA, USA) from greenhouse or garden-grown plants, which were produced from cuttings in summer 2010 from one of the female plants from the one known population in GA.

*R. michauxii* seeds (Seed Source 1) (148) from 13 female plants at the North Carolina Botanical Garden (Chapel Hill, NC, USA) were collected in 2005 and stored in the NC Botanical Garden Seed Banking Program at −20 °C. The seeds were provided to us in 2011.

*R. michauxii* seeds (Seed Source 2). With only two *R. michauxii* sites known in Georgia and both containing single sexes, rhizome transplants were taken from female plants at a site near Covington, GA, USA and from male plants at the Lower Broad River Wildlife Management Area near Elberton, GA, USA. Rhizomes collected in the spring were grown in a greenhouse at the Atlanta Botanical Garden in a potting mix consisting of five parts milled sphagnum moss, three parts perlite, two parts fir bark, two parts fine tree fern, two parts fine charcoal, and one part peat moss. Once established, rhizomes were transplanted to a sandy loam soil at the Smithgall Safeguarding Conservation Nursery near Gainesville, GA, USA. In 2010, rhizome transplants from clones of the female population grown at the Atlanta Botanical Garden (Atlanta, GA, USA) were planted adjacent to the natural male plants at the Broad River Wildlife Management Area (Elberton, GA, USA). In 2012, several female plants produced seeds that were collected in December.

*R. michauxii* seeds (Seed Source 3). Seeds from hand-pollination were collected from the Smithgall Nursery (Gainesville, GA, USA) in October 2013.

*R. michauxii* seeds (Seed Sources 4 and 5). Seeds were collected in September 2018 from the Lower Broad River Wildlife Management Area (Elberton, GA, USA). Source 4 seeds were collected from four infructescences from healthy-appearing female plants near the male plants. Source 5 seeds were collected from female plants located far from the male plants.

*R. michauxii* seeds (Source 6). In 2015, rooted-cutting-derived plants, grown at the Atlanta Botanical Gardens (Atlanta, GA, USA) from the male and female Georgia populations were placed in a rooftop garden at the Atlanta Zoo (Atlanta, GA, USA). These grew into a 9 m × 9 m area. Seeds were collected in September 2018.

The plant and seed sources used are summarized in Table 3. Since the descriptions in the table are abbreviated, the original descriptions are maintained above.

Experimental treatments were arranged in a completely randomized design. A replicate most often was a single seed per Petri plate or explant per container. Seeds were evaluated with scores of 1 for germination and 0 for no germination. Data were analyzed by the analysis of variance, and significant differences between the treatments were determined by the multiple-range test using Statgraphics Plus V4.0 (Manugistics, Rockville, MD, USA).

### 4.2. R. glabra Seed Sterilization and Germination Experiments

Four experiments were carried out, covering twelve scarification times and ranging from 0.5 to 8 h. Seeds were rinsed in tap water for 10 min, scarified by gently stirring in 7 mL of concentrated 18 M sulfuric acid (EM Science, Gibbstown, NJ, USA), followed by three rinses in sterilized room temperature water, each for 5 min. The number of seeds varied from 30 to 105 per scarification time for a total of 645 seeds tested. Scarified seeds were placed randomly onto three media, each with or without 0.5 mg/L BAP: ½ MS, MS, and WPM [42,43] with 3% sucrose, 100 mg/L myo-inositol, 0.50 mg/L thiamine HCl, 0.25 mg/L pyridoxine HCl, 0.25 mg/L nicotinic acid, and 1.0 mg/L glycine. The pH of media was adjusted to 5.7 and solidified with 4.5 g/L Phytagel. Media were autoclaved at 121 °C for 20 min. Since light improves germination of some *Rhus* species, the plates were wrapped in two layers of Parafilm and incubated under light at room temperature [44]: 24–25 °C with a 16/8-h (day/night) photoperiod of light supplied by cool white fluorescent lamps at an intensity of ~30 μmol m^−2^ s^−1^. Several seeds were removed from the acid during the treatment and the seed coat was observed.

With observations of negative effects of BAP in the germination medium and optimal scarification times appearing to occur between 5 and 8 h, the scarification time of 6 h was selected to compare the effects of several PGR-free media. One hundred and eighty *R. glabra* seeds were scarified in concentrated H_2_SO_4_ for 6 h and rinsed five times in sterile water, 5 min for each rinse. The floating seeds were discarded and the remaining 150 seeds were randomly placed onto three test germination media without PGRs: ½ MS, MS, and WPM.

### 4.3. R. michauxii Seed Scarification, Germination, and Seedling Establishment in Potting Mix

*R. michauxii* seeds (Seed Source 1) from the NC Botanical Garden were scarified for 6 h, as described above. The 148 seeds were placed individually on 7 mL of ½ MS germination medium contained in 60 × 15-mm Petri plates. The plates were wrapped with Parafilm and placed in a lighted culture room at 24–25 °C under a 16/8-h (day/night) photoperiod with light supplied by cool white fluorescent lamps at an intensity of approximately 30 μmol m^−2^ s^−1^. The germination responses were evaluated weekly.

Using the above procedure, three additional germination tests were run with four seed sources, as indicated below.

*R. michauxii* seeds (Source 2) consisting of 114 seeds from the Lower Broad River Wildlife Management Area were separated into those that sank or floated in distilled water. All of the seeds were scarified for 6 h, as indicated above. Seeds were divided amongst three treatments: Scarified seeds planted directly in the greenhouse potting mix; scarified seeds placed on the ½ MS and any seedlings were transferred to the greenhouse potting mix within several days of the radicle formation; and scarified seeds placed on ½ MS and seedlings grown for 1 month and then transferred to the potting mix. The greenhouse potting mix consisted of a mixture of five parts milled sphagnum moss, three parts perlite, two parts fir bark, two parts fine tree fern, two parts fine charcoal, and one part peat moss.

*R. michauxii* seeds (Sources 4, 5, and 6) that sank in distilled water were scarified for 6 h and placed on ½ MS medium. Any seedlings were transferred to the greenhouse potting mix within 48 h of visible radicle formation. The same potting mix that was used for Source 2 was used here, but without the fine tree fern. Seeds with a visible radicle were carefully removed from the agar, gently washed in running tap water, and planted in the potting mix in 9 cm pots in flats. Flats were covered with humidity domes. Domes were removed after about 2 weeks. Pots were watered every other day. Surviving plants were transferred to 15 cm pots containing the same potting mix after about 3 months.

### 4.4. R. glabra Shoot Culture Experiments

Three small tests were conducted to increase shoot production from axillary buds and the developing rhizome at the shoot base.

First experiment: 20 seedlings from scarified seeds were grown until six true leaves formed above the cotyledons. To test if apical dominance altered shoot formation, the plants were cut into two segments. The shoot tip portion with three leaves and the basal portion with three leaves and roots were placed onto 20 mL of MS medium with 0.05 mg/L BAP contained in Magenta boxes (Magenta, Chicago, IL, USA) and incubated under the same conditions as for germination. The resulting shoots were evaluated after 4 weeks of growth.

In the second experiment, 16 shoots were placed on the MS medium without PGRs. Eight shoots were grown at 24–25 °C in the light and eight were grown in the dark at 4 °C for 4 weeks, followed by 2 weeks in the light at 24–25 °C.

In a third experiment, thirty-four 2-week-old seedlings produced from 6 h scarified seeds germinated on ½ MS with roots removed were separated and placed with one half on MS + 2.5 g/L activated charcoal (AC, Sigma, St. Louis, MO, USA, catalogue no. C-4386) and another half on MS + 2.5 g/L AC + 0.05 mg/L BAP. Shoots were grown in the light for 4 weeks.

### 4.5. R. michauxii Bud Sterilization and Multiplication Experiments

Shoot tips and axillary buds from *R. michauxii* rooted cuttings grown in the greenhouse and outdoors were collected in October 2010. Three sterilization procedure modifications based on Ma et al. and Pullman et al. [45,46] varying in sterilization strength were tested: (1) 10 min Liqui-Nox/Tween 20, 30 min tap water, 10 min 20% H_2_O_2_, three 5 min sterile water washes; (2) 10 min Liqui-Nox/Tween 20, 30 min tap water, 30 s in ethanol, 5 min tap water rinse, 10 min 20% H_2_O_2_, three 5 min sterile water washes; (3) 10 min agitating in Liqui-Nox/Tween 20 (Sigma Aldrich, St Louis, MO, USA), 30 min under running tap water, 12.5 min agitation in 20% H_2_O_2_, three 5 min washes in sterile water while stirring. Due to the limited tissue, only 8–9 axillary buds and shoot tips were available per sterilization treatment. The ethanol soak in the second procedure was added to reduce the contamination associated with the pubescent stem and bud surface. The buds were divided amongst three media: 1/3 MS with 2.0 mg/L trans zeatin, ½ MS with 2.0 mg/L trans zeatin, and ½ MS with 0.5 mg/L BAP.

Sterilization of additional buds, collected in November, was attempted with modifications of the second procedure above. In one treatment, 15 s of ethanol dip replaced the 30 s dip. In another treatment, the ethanol dip was replaced with dissection of buds and shoot tips in 5% H_2_O_2_ with three 5 min rinses in sterile water. All of the tissues treated with these modifications were contaminated with fungus.

The few clean buds resulting from surface sterilization experiments were subcultured after 4 weeks to media containing ½ MS, MS or WPM with 0.5 mg/L BAP. After 2 weeks, if multiple buds were present, they were separated. In addition, all of the buds were moved to PGR-free MS or WPM to help the buds develop into shoots for 2 weeks. The resulting buds or shoots were subcultured to MS with 0.05 or 0.01 mg/L BAP for 6 weeks. The resulting shoots were subcultured approximately every 6 weeks to MS + 0.05 mg/L BAP.

### 4.6. R. michauxii Root Induction In Vitro and Acclimation to Soil

Twelve shoots from the mature female plant that had spontaneously formed roots in culture were transplanted to the greenhouse potting mix, consisting of a mixture of five parts milled sphagnum moss, three parts perlite, two parts fir bark, two parts fine tree fern, two parts fine charcoal, and one part peat moss. Six shoots had a normal appearance and six shoots had a hyperhydric appearance (Figure 8D).

### 4.7. R. glabra and R. michauxii Seed Cryopreservation in Liquid Nitrogen

*R. glabra* seed survival before and after rapid immersion in liquid nitrogen (LN) was tested by germinating seeds in ½ MS medium without PGRs. Freshly collected seeds were cleaned to remove the soft tissues and were placed in water. Four of the 50 seeds floated. The floating seeds were cut open and did not contain embryos. The remaining seeds were cleaned and the floaters were discarded. Ten seeds were placed in an oven for 24 h at 105 °C and the % moisture content on a dry weight basis was determined. One hundred seeds were divided into two lots of 50 and placed into 2-mL Nalgene cryogenic storage vials (Thermo Scientific, Waltham, MA, USA). One vial was rapidly immersed in LN. After 98 h, the vial was removed from LN and rewarmed in a 37 °C water bath for 1–2 min. The additional 50 seeds were held at room temperature. Both lots of seeds were scarified for 6 h in concentrated sulfuric acid and rinsed in sterile water, as previously described.

*R. michauxii* seed survival after cryopreservation was tested by germinating seeds in vivo and in vitro. Sixty seeds from *R. michauxii* (Source 3) were placed in a 2-mL Nalgene cryogenic storage vial and rapidly immersed in LN. After 48 h, the vial was removed from LN and rewarmed in a 37 °C water bath for 1–2 min. Sixty additional seeds were held at room temperature. Both lots of seeds were scarified for 6 h, as described above. Half of each lot was placed onto three media with 10 seeds per medium: ½ MS, ½ MS with KH_2_PO_4_ reduced by half (to better simulate soil conditions where *R. michauxii* grows), and ½ MS with 0.05 mg/L BAP, for germination in vitro. The remaining 30 seeds for each lot were planted in the greenhouse mix, as described above.

## Figures and Tables

**Table 1:** *Rhus michauxii* seed sources scarified and tested for germination in 2018.

Seed Source and Origin	No. of Seeds Collected	Seeds Sinking (%)	No. (%) of Germinants after 1 Month	No. (%) of Contaminated Seeds after 1 Month	Seedlings Planted in Potting Soil	Seedlings Surviving after 1 Month
Source 4—LBRWMA, females near the male plants	515	10.3	22/53 (39.6)	14/53 (26.4)	16	11
Source 5—LBRWMA, females away from the male plants	243	0.4	0/1 (0)	0/1 (0)	0	0
Source 6—Zoo Atlanta rooftop garden	2600	0.3	1/8 (12.5)	1/8 (12.5)	1	1

**Table 2:** Recommended steps for *Rhus michauxii* seed cryopreservation, germination, and establishment of in vitro germinated seedlings in the potting mix.

Seed Cryopreservation
(1) Select seeds sinking in distilled water, dry seeds; (2) Determine if seed moisture content is below 14% and further dry, if needed; (3) Place seeds in cryogenic storage vials; (4) Rapidly immerse vials in liquid nitrogen (LN); (5) Remove vials from LN and re-warm in a 37 °C water bath for 1–2 min; (6) Germinate seeds in vitro.
Seed germination in vitro (production of seedlings for planting or shoots for micropropagation)
(1) Scarify non-cryopreserved or cryopreserved seeds in concentrated sulfuric acid for 6 h; (2) Rinse seeds in sterile distilled water three times; (3) Place on the ½ MS medium without PGRs in sterile containers; (4) Incubate in light at room temperature; (5) Remove germinants for planting or micropropagation.
Germinant/seedling establishment in the potting mix
(1) Select germinating seeds when the radicle becomes visible; (2) Carefully remove from agar, rinse in tap water, and plant in the potting mix; (3) Cover with humidity domes for about 2 weeks. Water every other day. Transfer to larger pots after about 3 months.

**Table 3:** Bud and seed sources for *Rhus glabra* and *Rhus michauxii*.

Species	Plant Part and Source	Collection Site	Collection Time	Pollination	Notes
*R. glabra*	seeds	SSCN	Oct 2010	Natural	Wild plants
*R. michauxii*	buds	ABG	Oct, Nov 2010		Buds and shoot tips from cuttings of a female plant from the natural population at Covington, GA
*R. michauxii*	Seeds 1	NCBG	2005	Natural	Seeds were stored in the NCBG Seed Banking Program at −18 °C
*R. michauxii*	Seeds 2	LBRWMA	Dec 2012	Natural	From female cuttings planted near natural male plants at the LBRWMA
*R. michauxii*	Seeds 3	SSCN	Oct 2013	Hand-pollinated	From female cuttings mixed with male cuttings planted in the SSCN
*R. michauxii*	Seeds 4	LBRWMA	Sept 2018	Natural	Seeds collected from cuttings of females planted near natural male plants at the LBRWMA
*R. michauxii*	Seeds 5	LBRWMA	Sept 2018	Natural	Seeds collected from females away from male plants at the LBRWMA
*R. michauxii*	Seeds 6	Atlanta Zoo	Sept 2018	Natural	Seeds collected from female cuttings planted in 2015 with male cuttings

SSCN: Smithgall Safeguarding Conservation Nursery near Gainesville, GA; ABG: Atlanta Botanical Garden; NCBG: North Carolina Botanical Garden; LBRWMA: Lower Broad River Wildlife Management Area.

## Data Availability

Data is contained within the article.
